# High rates of organic carbon processing in the hyporheic zone of intermittent streams

**DOI:** 10.1038/s41598-017-12957-5

**Published:** 2017-10-16

**Authors:** Ryan M. Burrows, Helen Rutlidge, Nick R. Bond, Stefan M. Eberhard, Alexandra Auhl, Martin S. Andersen, Dominic G. Valdez, Mark J. Kennard

**Affiliations:** 1 Australian Rivers Institute, Griffith University, Nathan, Queensland Australia; 2 Connected Waters Initiative Research Centre, UNSW Sydney, Australia; 3 School of Civil and Environmental Engineering, UNSW Sydney, Australia; 4 Murray-Darling Freshwater Research Centre, La Trobe University, Wodonga, Australia; 5 Subterranean Ecology Pty Ltd, Coningham, Tasmania Australia; 6 School of Biological, Earth and Environmental Sciences, UNSW Sydney, Australia

## Abstract

Organic carbon cycling is a fundamental process that underpins energy transfer through the biosphere. However, little is known about the rates of particulate organic carbon processing in the hyporheic zone of intermittent streams, which is often the only wetted environment remaining when surface flows cease. We used leaf litter and cotton decomposition assays, as well as rates of microbial respiration, to quantify rates of organic carbon processing in surface and hyporheic environments of intermittent and perennial streams under a range of substrate saturation conditions. Leaf litter processing was 48% greater, and cotton processing 124% greater, in the hyporheic zone compared to surface environments when calculated over multiple substrate saturation conditions. Processing was also greater in more saturated surface environments (i.e. pools). Further, rates of microbial respiration on incubated substrates in the hyporheic zone were similar to, or greater than, rates in surface environments. Our results highlight that intermittent streams are important locations for particulate organic carbon processing and that the hyporheic zone sustains this fundamental process even without surface flow. Not accounting for carbon processing in the hyporheic zone of intermittent streams may lead to an underestimation of its local ecological significance and collective contribution to landscape carbon processes.

## Introduction

The processing of organic carbon (C) is a fundamental ecological process that underpins energy transfer throughout the biosphere. This process is particularly important in inland waters which, although covering less than 1% of the Earth’s surface, transport, mineralise, and bury a similar magnitude of C (~2.9 Pg C yr^−1^)^1^ as the entire terrestrial sink for anthropogenic emissions (2.8 Pg C yr^−1^)^2,3^. The disproportionally high rates of C processing, storage, and export in inland waters can influence the C balance of the landscapes they drain and, as a result, are an important dynamic component of the Earth’s C cycle^3–5^. Resolving the dominant factors driving the spatial and temporal patterns of C dynamics in inland waters has thus emerged as an important challenge to understanding how the global C cycle will respond to climate change and other anthropogenic stressors. Meeting this challenge is particularly important in intermittent streams, which constitute 69% of first order streams below 60 °N^4^ and which are expected to increase in prevalence over the next century due to climate change and the abstraction of groundwater^6,7^. Despite their increasing prevalence, we have a very limited understanding of intermittent stream contributions to landscape particulate organic C processing.

The physical and biological factors mediating particulate C processing, such as leaching and the foraging activities of macroinvertebrate detritivores, are largely dependent on the presence of water. Thus, cycles of wetting and drying govern variation in rates of particulate organic C processing in surface environments of intermittent streams^8–10^. Despite the pervasive role that surface hydrological conditions have on rates of particulate C processing in surface environments, little is known about the spatial and temporal patterns of particulate C processing in subsurface, or hyporheic, environments of intermittent streams. The hyporheic zone consists of saturated sediments associated with the active channel and riparian zone of streams and rivers in which surface water and groundwater mix^11^, and is often the only wetted environment remaining during drying cycles in intermittent and ephemeral streams^12^.

The hyporheic zone is considered a ‘permanent control point’ in the landscape for ecological and biogeochemical processes^13^, because permanently connected sub-surface flow-paths often provide the environmental conditions necessary (i.e. moisture, organic substrates, stable water temperature) for sustained and high rates of many processes, including nitrogen cycling^14^ and metabolism of dissolved organic C^15,16^. However, in perennial streams, there is no evidence that the hyporheic zone supports comparatively greater rates of particulate organic C processing than wetted surface environments^17–20^. One exception involved the subsurface breakdown rates of alder leaves, which were greater than surface patches in the parafluvial zone of a perennial mountainous stream, due to the surficial drying of the parafluvial zone during summer^21^. Given that the hyporheic zone of intermittent streams often remains saturated during dry periods^12^, and particulate organic C processing in surface environments is constrained by frequent drying^8–10^, the hyporheic zone may support comparatively higher rates of particulate organic C processing relative to surface environments when examined across wet and dry phases.

In this study, we quantify how organic C processing varies in surface and hyporheic environments of intermittent streams. We answer this question using two powerful and complementary experimental measures of organic C processing (leaf litter processing and cotton strip or cellulose processing) in different surface channel units (gravel bars, pools, and riffles) and in the hyporheic zone in eight streams spanning two geographic regions in eastern Australia. Additionally, we assessed how rates of microbial respiration of biofilms colonising leaf and cellulose substrates vary in surface and hyporheic environments. We hypothesised that rates of organic C processing would be consistently greater in hyporheic sediments compared to paired surface channel units of intermittent streams because the hyporheic zone remains saturated for longer. Ultimately, understanding the rates of, and controls on, organic C processing in the hyporheic zone of intermittent streams is critical to understanding and predicting how potential changes in intermittency and baseflow will affect stream energy transfer as well as the broader C balance of landscapes.

## Results

Leaf decay rates were consistently greater in the hyporheic zone than in all surface environments, except in perennial riffles and pools (Table 1). Pooling sites, regions, and incubation periods, decay rates were less variable in the hyporheic zone (coefficient of variation: CV = 7.2) than in intermittent and perennial gravel bars (CV = 32.0), intermittent pools (CV = 27.4), and intermittent riffles (CV = 30.2), but slightly more variable than perennial pools (CV = 4.14) and riffles (CV = 6.14). Leaf litter processing was predominantly mediated by microbial organisms, with leaf % mass loss similar in both fine- (excludes macroinvertebrates) and coarse-mesh bags in surface (Linear mixed-effect model or LMM: *P* = 0.71, *n* = 470) and hyporheic (LMM: *P* = 0.81, *n* = 317) environments. Paired substrate patches were significantly wetter in the hyporheic zone than the surface in both the leaf litter (hyporheic mean = 3.4, surface mean = 1.8; *P* < 0.001, *n* = 357) and cotton strip (hyporheic mean = 4.6, surface mean = 3.1; *P* < 0.001, *n* = 571) incubations (see Supplementary Figs S1 and S2). Water samples collected from the hyporheic zone in both regions tended to be oxic, except for Reynolds Creek. Hyporheic dissolved oxygen (DO) was consistently highest in the hyporheic zone of the perennial study stream (Table 2), though DO levels fluctuated with occasional high levels in Wild Cattle Creek. pH was circumneutral at all sites and dissolved organic C (DOC) concentrations were, on average, low ( < 4.5 mg C L^−1^). Mean NH_4_^+^ concentrations were generally low ( < 14 µg N L^−1^) but were most elevated at Horsearm Creek (Table 2). Mean NO_3_^−^ concentrations were below 22 µg N L^−1^ in the south-eastern Queensland (QLD) streams and at Maules Creek, but were elevated at Middle Creek (mean = 170 µg N L^−1^) and Horsearm Creek (mean = 510 µg N L^−1^) (Table 2). Soluble reactive phosphorus (SRP) was elevated in the northern New South Wales (NSW) streams (mean = 198 µg P L^−1^) but was, on average, below 53 µg P L^−1^ in the south-eastern QLD streams (Table 2). Mean pool and riffle depth was highly variable among and within (relatively large standard deviations) study streams (Table 2). Surface water was enriched in ^222^Rn in all study sites (Table 2).

**Table 1:** The mean ± standard deviation (s.d.), if available, and range (minimum − maximum, if available) in leaf litter decay rates (*k*) in surface and hyporheic environments from our study, and other comparable studies, in intermittent and perennial streams.

Study	Flow regime	Climate	Channel unit	Mesh size	Species	*k* (mean ± s.d.)	*k* (range)
1	Int	HS	Hyporheic	Fine and coarse	*Eucalyptus tereticornis* and *E. camaldulensis*	0.058 ± 0.004	0.048–0.071
1	Int	HS	Gravel bar	Fine and coarse	*E. tereticornis* and *E. camaldulensis*	0.036 ± 0.016	0.000–0.061
1	Int	HS	Pool	Fine and coarse	*E. tereticornis* and *E. camaldulensis*	0.054 ± 0.015	0.000–0.070
1	Int	HS	Riffle	Fine and coarse	*E. tereticornis* and *E. camaldulensis*	0.049 ± 0.015	0.000–0.072
1	Per	HS	Hyporheic	Fine and coarse	*E. camaldulensis*	0.056 ± 0.002	0.051–0.064
1	Per	HS	Gravel bar	Fine and coarse	*E. camaldulensis*	0.036 ± 0.012	0.003–0.049
1	Per	HS	Pool	Fine and coarse	*E. camaldulensis*	0.057 ± 0.002	0.053–0.061
1	Per	HS	Riffle	Fine and coarse	*E. camaldulensis*	0.061 ± 0.004	0.054–0.067
2	Int	Tem	Riffle	Coarse (6mm)	*Alnus glutinosa*	0.0036 ± 0.0037	0.0005–0.012
3	Int	Med	Isolated pools	Coarse (5mm)	*Populus nigra*	0.044 ± 0.005	NA
3	Int	Med	Moist sediment	Coarse (5mm)	*P. nigra*	0.013 ± 0.001	NA
3	Int	Med	Dry sediment	Coarse (5mm)	*P. nigra*	0.009 ± 0.002	NA
3	Per	Med	Running water	Coarse (5mm)	*P. nigra*	0.053 ± 0.003	NA
4	Per	Med	Running water	Fine (0.01mm)	*E. marginata*	0.0012–0.0015	NA
4	Per	Med	Running water	Coarse (3.4mm)	*E. marginata*	0.0014–0.0020	NA
5	Per	Mon	Riffle	Coarse (5mm)	*A. glutinosa*	0.0292	NA
5	Per	Mon	Hyporheic	Coarse (5mm)	*A. glutinosa*	0.0103	NA
6	Per	Mon	Wet channel - surface	Coarse (5mm)	*A. glutinosa*	0.0125 (est.)	NA
6	Per	Mon	Wet channel - hyporheic	Coarse (5mm)	*A. glutinosa*	0.0110 (est.)	NA
6	Per	Mon	Parafluvial - surface	Coarse (5mm)	*A. glutinosa*	0.0055 (est.)	NA
6	Per	Mon	Parafluvial - hyporheic	Coarse (5mm)	*A. glutinosa*	0.0115 (est.)	NA

Results are for combined fine- and coarse-mesh leaf litter bags for our study. If mean values were not stated in the text of other studies we estimated (est.) values from the plots presented. Flow regime: Int = Intermittent; Per = Perennial. Climate: HS = Humid subtropical; Tem = Temperate; Med = Mediterranean; Mon = Montane. Study: 1 = Present study; 2 = Datry and others (2011); 3 = Abril and others (2016); 4 = Bunn (1988); 5 = Cornut and others (2010); 6 = Solagaistua and others (2016).

**Table 2:** Environmental characteristics of the eight study streams in south-eastern Queensland (QLD) and northern New South Wales (NSW).

Environmental characteristic	Stream							
	Bremer River	Warrill Creek	Coulson Creek	Wild Cattle Creek	Reynolds Creek	Middle Creek	Horsearm Creek	Maules Creek
Flow regime	Intermittent	Intermittent	Intermittent	Intermittent	Intermittent	Intermittent	Perennial	Intermittent
Catchment area (km^2^)	5.97	19.9	15.6	45.3	38.2	106	159	390
Reach elevation (m asl)	284	202	240	165	172	336	293	287
Hyporheic exchange	Downwelling	Neutral to downwelling	Neutral	Neutral to downwelling	Neutral	Downwelling	Upwelling	Neutral to downwelling
Hyporheic DO (mg L^−1^)	1.51 ± 1.4	2.91 ± 0.3	1.80 ± 2.4	6.79 ± 7.5	0.09 ± 0.1	1.22 ± 1.7	3.09 ± 2.0	0.46 ± 1.0
Surface DO (mg L^−1^)	4.62 ± 2.2	1.88 ± 1.0	3.84 ± 2.2	7.88 ± 10	9.95 ± 2.7	3.98 ± 2.1	5.80 ± 0.7	7.34 ± 3.8
^222^Rn (Bq m^−3^)	2951 ± 953	2041 ± 743	3346 ± 1260	2624 ± 129	3319 ± 777	Not measured	774 ± 473	1348 ± 1059
Water temperature (°C)	19.9 ± 2.1	22.9 ± 1.5	22.9 ± 2.5	21.9 ± 1.7	21.6 ± 1.9	15.8 ± 3.8	15.9 ± 2.8	20.8 ± 7.7
NH_4_^+^ (µg N L^−1^)	4.74 ± 3.8	2.96 ± 3.1	8.11 ± 7.4	5.80 ± 6.5	12.2 ± 11	1.13 ± 1.8	31.4 ± 63	13.4 ± 17
NO_3_^−^ (µg N L^−1^)	21.7 ± 19.0	9.49 ± 7.2	18.9 ± 16	9.08 ± 6.0	3.18 ± 4.4	170 ± 260	510 ± 630	20.9 ± 28
SRP (µg P L^−1^)	52.8 ± 18	43.0 ± 12	15.1 ± 3.9	11.6 ± 2.2	14.8 ± 5.2	260 ± 130	222 ± 230	111 ± 220
DOC (mg C L^−1^)	1.54 ± 0.55	1.27 ± 0.22	1.43 ± 0.19	1.58 ± 0.96	1.55 ± 0.22	4.33 ± 4.6	1.84 ± 3.2	1.78 ± 1.2
pH	7.49 ± 0.64	7.75 ± 0.53	7.27 ± 0.53	7.43 ± 0.68	7.54 ± 0.61	6.69 ± 0.51	7.09 ± 0.27	7.26 ± 0.59
Pool depth (cm)	32.9 ± 14	24.8 ± 14	21.8 ± 16	44.7 ± 22	36.5 ± 21	9.05 ± 13	18.7 ± 5.7	11.6 ± 36
Riffle depth (cm)	4.79 ± 4.5	6.91 ± 4.5	2.60 ± 1.5	10.7 ± 6.4	11.6 ± 10.5	0.400 ± 1.6	7.00 ± 3.9	2.58 ± 7.1

The mean ± standard deviation is given for hyporheic dissolved oxygen (DO) as well as surface DO, radon gas (^222^Rn), water temperature, ammonium (NH_4_^+^), nitrate (NO_3_^−^), soluble reactive phosphorus (SRP), dissolved organic carbon (DOC), pH, as well as pool and riffle depth during observed water presence.

### Greater organic C processing in the hyporheic zone

**Figure 1:**
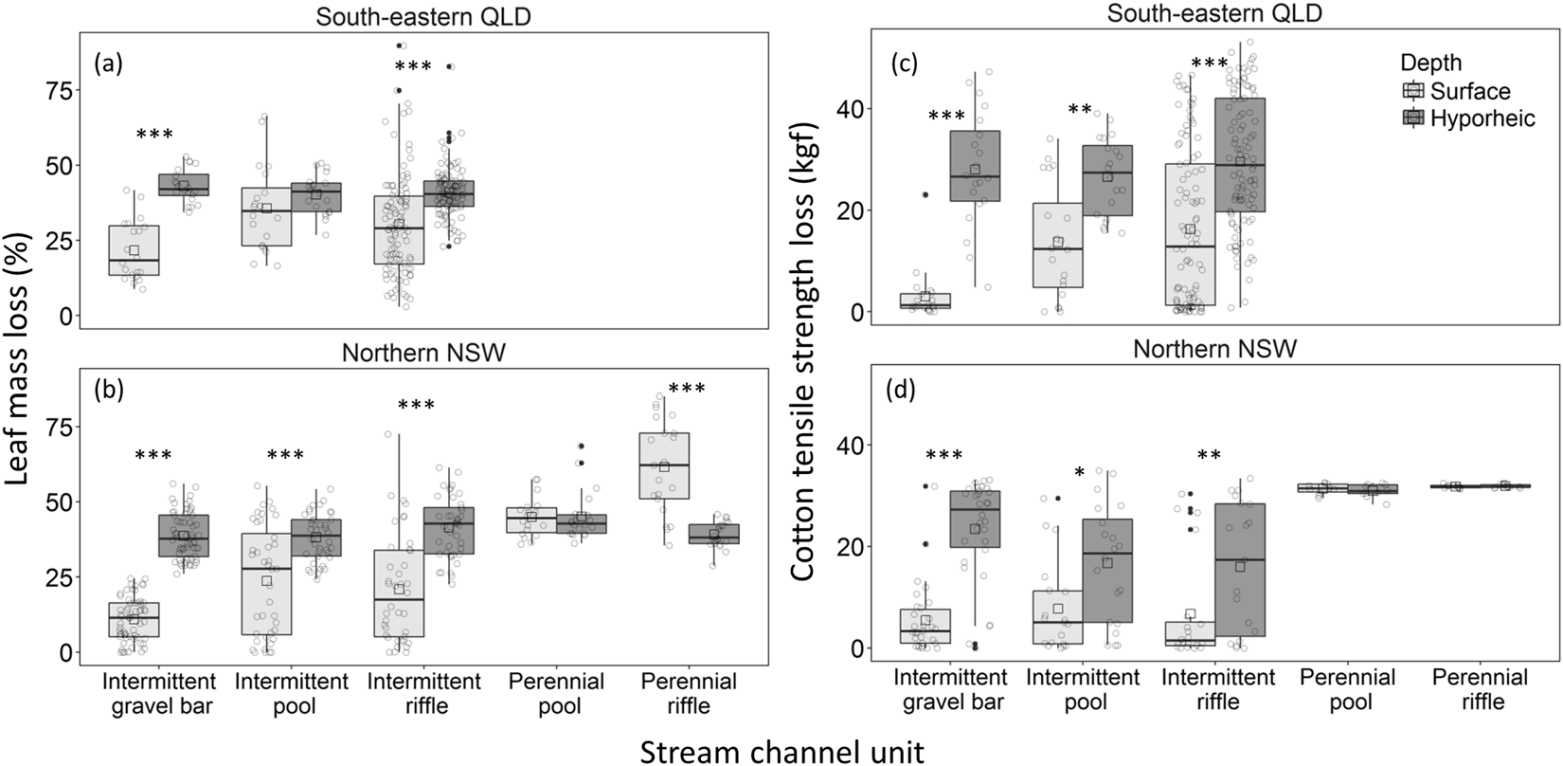
Box-whisker plots showing variation in (**a**,**b**) percent mass loss of leaf litter in fine- and coarse-mesh litter bags (data pooled) and (**c**,**d**) cotton tensile strength loss (kilogram-force or kgf) of cotton strips among surface and hyporheic patches of the dominant channel units of intermittent and perennial streams (gravel bars, pools, and riffles) within south-eastern Queensland (QLD) and northern New South Wales (NSW), Australia. The lines at the top, middle and bottom of each box represent the 75^th^ percentile, median and 25^th^ percentile of values, respectively. Vertical bars (whiskers) represent minimum and maximum values excluding outliers (solid dots), mean values are represented by hollow squares and individual observations as hollow circles. An asterisk indicates significant differences among surface and hyporheic samples with *P < 0.05, **P < 0.01 or ***P < 0.001 (determined using linear mixed-effect models – see ‘Statistical analyses’ section).

Overall, rates of leaf litter (Fig. 1a,b) and cotton (Fig. 1c,d) processing were considerably greater (48% and 124%, respectively) in hyporheic zone of intermittent streams compared to surface patches when calculated across study regions, sites, and incubation periods. In both regions, % leaf-mass loss was significantly greater (all terms; *P* < 0.01) in the hyporheic zone than paired surface patches in gravel bars and intermittent riffles (Fig. 1a,b). In particular, % leaf-mass loss in gravel bars was 2 and 3.6 times greater in the hyporheic zone compared to surface patches for the QLD (*P* < 0.001, *n* = 112) and NSW (*P* < 0.001, *n* = 120) regions, respectively (Fig. 1a,b). In intermittent pools, % leaf-mass loss in the hyporheic zone was only greater than paired surface patches in the NSW region (*P* < 0.001, *n* = 78), with no overall difference among surface and hyporheic patches in QLD (*P* = 0.49, *n* = 120) (Fig. 1a,b). Further data investigation, however, revealed some incubation-specific patterns in % leaf-mass loss, with mass loss in both regions greater in the hyporheic zone compared to paired surface patches of intermittent pools during the second, drier incubation period (QLD, *P* < 0.001, *n* = 60; NSW, *P* < 0.001, *n* = 40). In perennial pools, % leaf-mass loss was similar among paired surface and hyporheic patches (*P* = 1.0, *n* = 38) but was 1.6 times greater in surface compared to hyporheic patches for perennial riffles (*P* < 0.001, *n* = 40) (Fig. 1b).

Cotton processing was significantly greater in the hyporheic zone than paired surface patches in all intermittent channel unit types (Fig. 1c,d). Differences were greatest in gravel bars, with 840% and 330% more cotton tensile strength loss (CTSL) in the hyporheic zone than paired surface patches within the QLD (*P* < 0.001, *n* = 40) and NSW (*P* < 0.001, *n* = 60) regions, respectively (Fig. 1c,d). CTSL was 1.9 and 2.2 times greater in the hyporheic zone of intermittent pools than surface patches within the QLD (*P* < 0.01, *n* = 40) and NSW (*P* < 0.05, *n* = 40) regions, respectively (Fig. 1c,d); whereas in the perennial stream, mean values for CTSL were similar among paired surface and hyporheic patches (*P* = 1.0, *n* = 20) (Fig. 1d). For intermittent riffles, rates of CTSL were 1.8 and 2.4 times greater in the hyporheic zone than in paired surface patches within the QLD (*P* < 0.001, *n* = 198) and NSW (*P* < 0.01, *n* = 38) regions, respectively, but was similar between surface and hyporheic patches in the perennial stream (*P* = 1.0, *n* = 18) (Fig. 1c,d).

**Figure 2:**
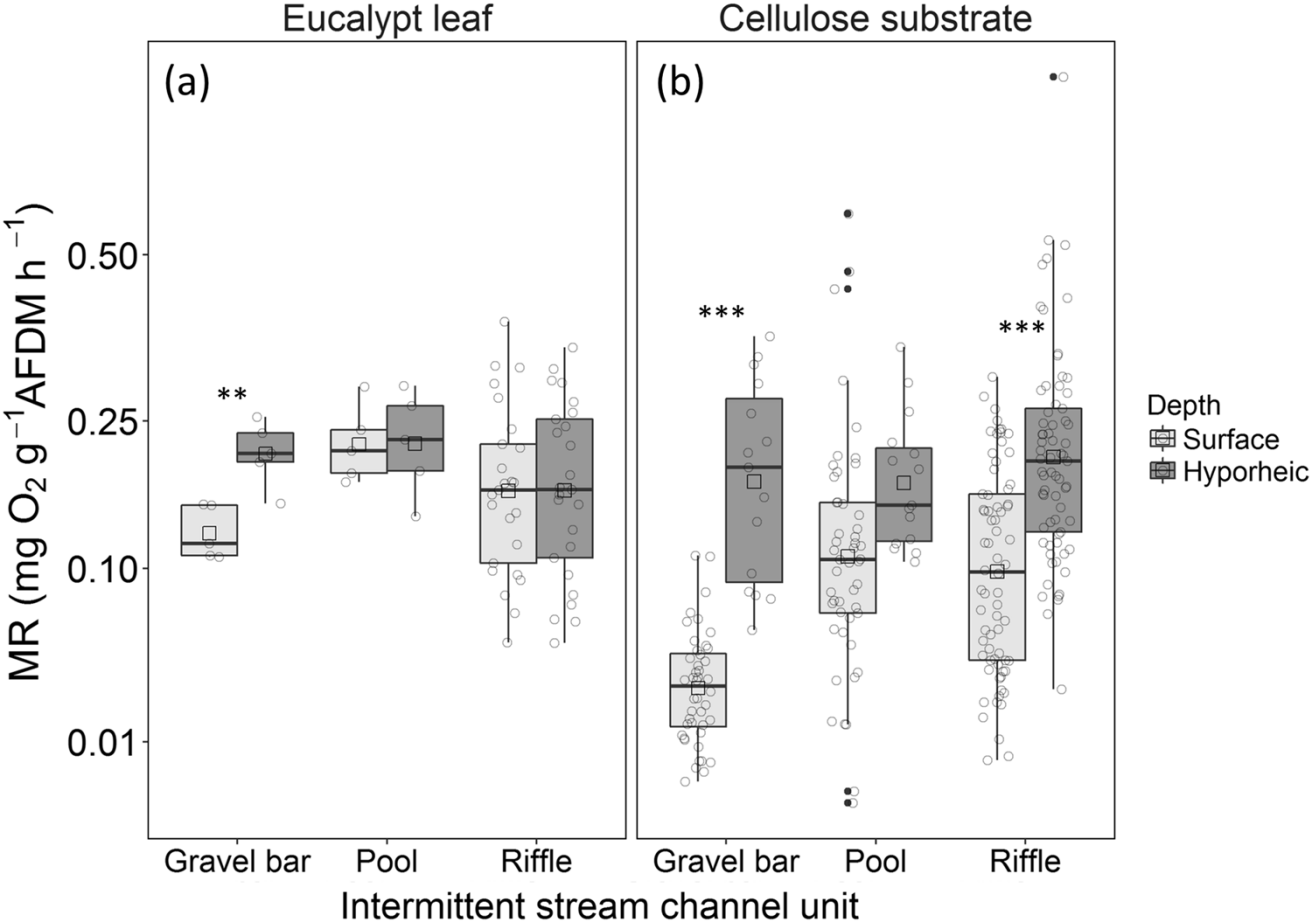
Box-whisker plots showing variation in rates of microbial respiration (MR) measured on (**a**) eucalypt leaves and (**b**) cellulose substrates incubated in surface and hyporheic patches of the dominant channel units of intermittent streams (gravel bars, pools, and riffles) in south-eastern Queensland. MR is represented per milligram of O_2_ consumed per gram of substrate ash-free dry-mass (AFDM) per hour. The lines at the top, middle and bottom of each box represent the 75^th^ percentile, median and 25^th^ percentile of values, respectively. Vertical bars (whiskers) represent minimum and maximum values excluding outliers (solid dots), mean values are represented by hollow squares and individual observations as hollow circles. An asterisk indicates significant differences among surface and hyporheic samples with **P < 0.01 or ***P < 0.001 (determined using linear mixed-effect models – see ‘Statistical analyses’ section).

Microbial respiration (MR) of biofilms colonising leaf material was greater in the hyporheic zone than surface patches of gravel bars, but there were no differences between surface and hyporheic patches for pools and riffles during this incubation period (Fig. 2). Rates of MR on artificial cellulose substrates were significantly greater in the hyporheic zone compared to surface patches of gravel bars and riffles, but not pools, in all three incubations periods (Fig. 2). Rates of MR were not significantly different among artificial cellulose substrates and eucalypt leaves on all channel units in the hyporheic zone (pools, *P* = 0.9; riffles, *P* = 0.09; gravel bars, *P* = 0.55) and also in surface patches of riffles (*P* = 0.4) and pools (*P* = 0.9). However, rates of MR were greater on eucalypt leaves than artificial cellulose substrates in surface patches of gravel bars (*P* = 0.02).

### Influence of substrate saturation and water temperature on organic C processing

**Figure 3:**
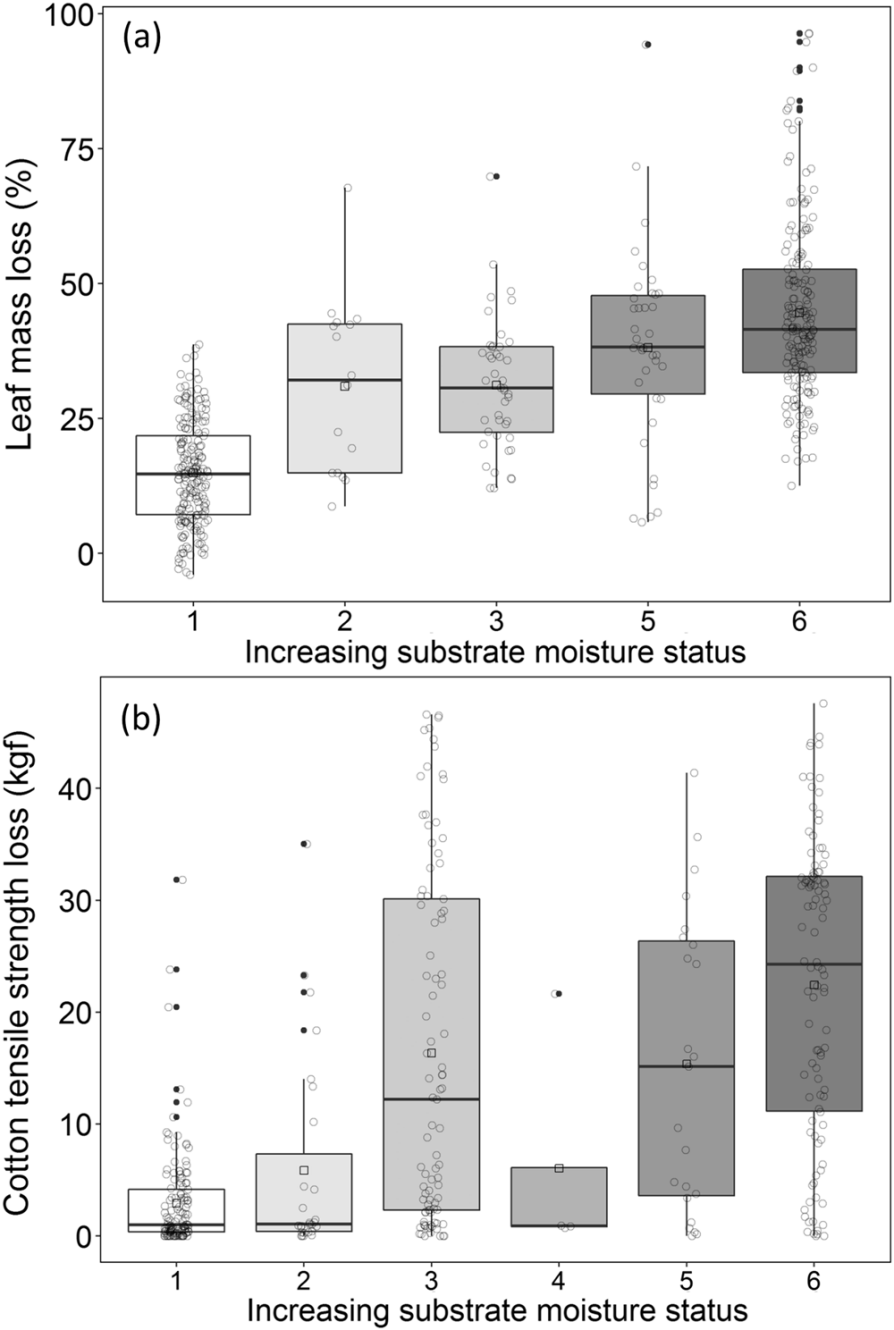
Box-whisker plots displaying variation in surface values for (**a**) % leaf mass loss and (**b**) cotton tensile strength loss (CTSL; kilogram-force﻿ or﻿ kgf) in six categories of substrate moisture status. Moisture status categories: dry-dry = 1; dry-moist = 2; moist-dry = 2; dry-saturated = 3; saturated-dry = 3; moist-moist = 4; moist-wet = 5; wet-moist = 5; wet-wet = 6. Greater CTSL values represent more cellulose decomposition than values closer to zero. The lines at the top, middle and bottom of each box represent the 75^th^ percentile, median and 25^th^ percentile of values, respectively. Vertical bars (whiskers) represent minimum and maximum values excluding outliers (solid dots), mean values are represented by hollow squares and individual observations as hollow circles.

Leaf litter % mass loss (*P* < 0.0001, *n* = 469) and CTSL (*P* < 0.0001, *n* = 381) were greater in surface patches subject to more saturated conditions (Fig. 3). Each increase in the substrate moisture category (i.e. from category 1 to 2) led to a 5% increase in % leaf mass loss over the incubation period, with 52% more litter processing in the wettest moisture category (category 6) than in the driest moisture category (category 1). For cotton strips, each increase in substrate moisture category led to an 8% increase in tensile-strength loss over the incubation period, with CTSL in the wettest moisture category (category 6) 315% greater, on average, than in the driest moisture category (category 1). Neither leaf litter % mass loss (*P* = 0.22, *n* = 317) nor CTSL (*P* = 0.89, *n* = 228) were significantly related to variation in substrate moisture in the hyporheic zone. Rates of MR on leaf (*P* = 0.0003, *n* = 34) and cellulose substrates (*P* < 0.0001, *n* = 148) were greater in more saturated surface patches, but showed no relationship with substrate moisture on either leaf (*P* = 0.11, *n* = 34) or cellulose substrates (*P* = 0.35, *n* = 99) in the hyporheic zone. The % leaf-mass loss, of leaf litter bags saturated for the entire incubation period, was not correlated with mean (*R* ^2^ = 0.00, *P* = 0.6, *n* = 52), median (*R* ^2^ = 0.01, *P* = 0.3, *n* = 52) or the CV (*R* ^2^ = 0.04, *P* = 0.2, *n* = 52) of water temperature, but showed a weak, positive correlation with the minimum recorded water temperature (*R* ^2^ = 0.15, *P* = 0.004, *n* = 52) (Supplementary Fig. S3). Similar patterns were observed for leaf decay rate.

## Discussion

The breakdown of organic C is a fundamental ecosystem process in streams and rivers that can influence the C dynamics of the landscapes they drain^3–5^. Organic C processing in the hyporheic zone of our intermittent study streams was consistently higher than in paired surface patches, even when surface flow ceased. Most notably, rates of cotton processing were 840% greater in the hyporheic zone of gravel bars compared to paired surface patches in one study region. Furthermore, rates of biofilm MR on leaf and cellulose substrates in the hyporheic zone were greater than, or similar to, surface patches of gravel bars, riffles, and pools. Overall, the findings generally confirm our hypothesis of greater rates of organic C processing in the hyporheic zone compared to surface patches, and suggest that the hyporheic zone of gravel-dominated intermittent streams may be considered an ecosystem control point in the landscape for particulate organic C processes. Given that intermittent streams are widespread^4^, and their prevalence is expected to increase over the next century^6,7^, failure to account for C processes in the hyporheic zone of many intermittent streams may lead to an underestimation of their local ecological significance and collective contribution to landscape C processing.

The hyporheic zone has been demonstrated to have near constant environmental conditions appropriate for sustaining high rates of many biogeochemical and ecological processes compared to surrounding environments^15,22,23^. The higher rates of leaf and cotton (i.e. particulate organic C) processing in the hyporheic zone compared to surface channel units in our intermittent streams corroborates this notion. Applying a nuanced ecological framework for describing the hot spot and hot moment concept, as outlined in ref.^13^, we propose that the hyporheic zone of many intermittent streams should be termed an activated ecosystem control point for particulate organic C processing. This is because disproportionately higher rates of particulate organic C processing in the hyporheic zone, compared to surrounding surface environments, is likely ‘activated’ only when particulate organic matter is available. Further, our findings indicate that the hyporheic zone of perennial streams is not an ecosystem control point for particulate organic C processing because rates of C processing were similar or greater in wetted surface patches (i.e. riffles and pools) compared to paired hyporheic patches – a trend supported by previous research in perennial systems^17–20^.

**Figure 4:**
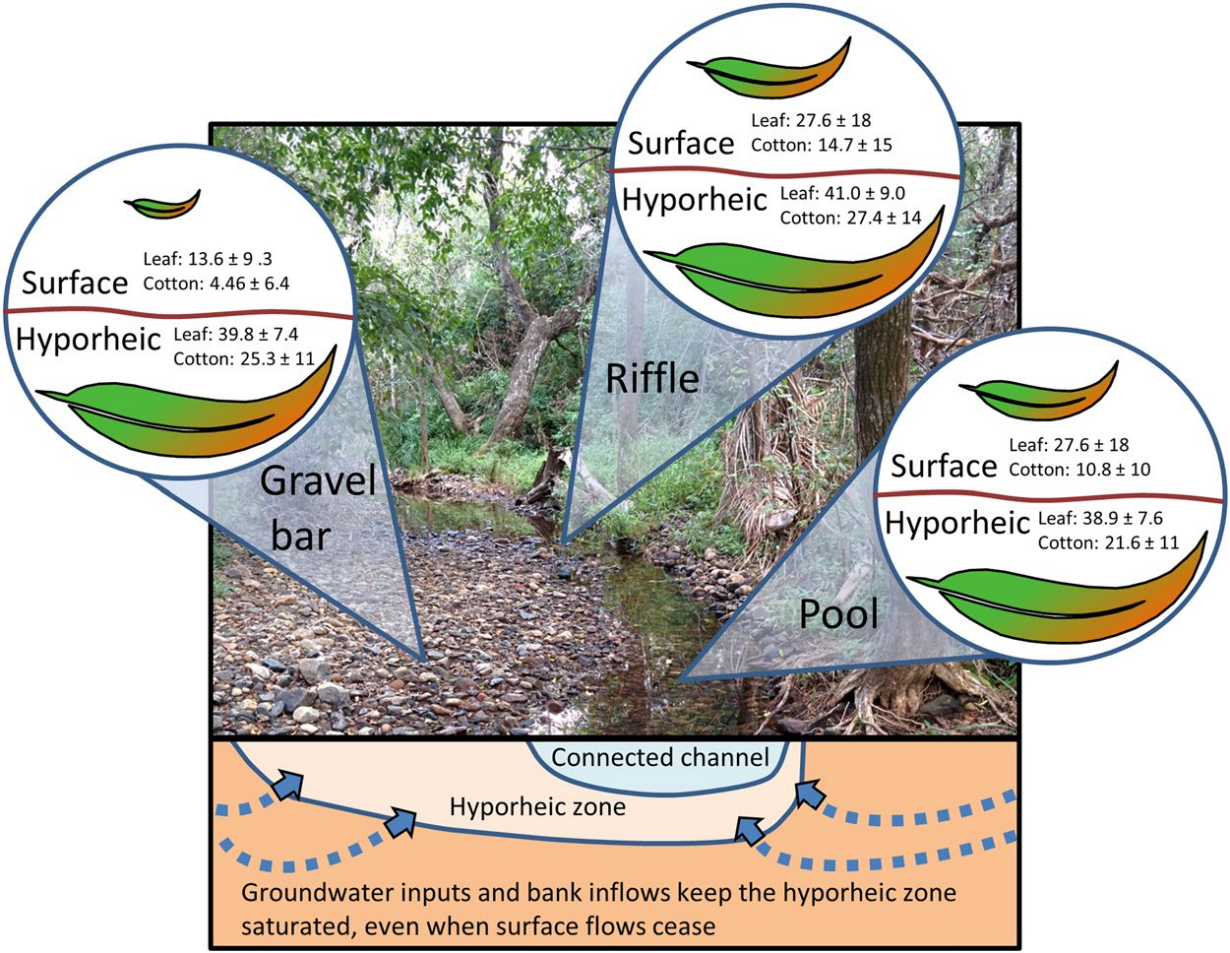
Schematic summarising variation in the rates of particulate organic carbon processing among intermittent stream environments and the role of groundwater inputs (indicated with dashed blue arrows) in sustaining the saturated hyporheic zone. The mean ± standard deviation of % leaf mass loss (‘Leaf’) and cotton tensile strength loss (kilogram-force or kgf) (‘Cotton’) is shown for the surface patches of gravel bars, riffles, and pools, as well as for the hyporheic zone, for the intermittent study streams. Leaf and cotton substrates were incubated during multiple incubation periods that encompassed natural variability in surface flow. The size of each leaf is proportional to the degree of particulate organic carbon processing.

Organic C processing was predominately mediated by microbial processes and not by macroinvertebrate consumption (as evidenced by similar C processing rates in both fine- and coarse-mesh litter bags) in both surface and hyporheic environments. Moreover, the hyporheic zone was consistently more saturated than corresponding surface patches and rates of surface C processing were positively associated with substrate saturation. Together, these results indicate that continued substrate saturation was a key factor enabling the high rates of microbial organic C processing in the hyporheic zone. Groundwater inputs likely sustained a saturated hyporheic zone, because elevated radon values, as we recorded in our study streams, are indicative of high groundwater-surface water connectivity^24^. Consequently, we suggest that saturated sediments from groundwater inputs was a key factor enabling elevated rates of organic C processing in the hyporheic zone of our intermittent study streams (Fig. 4).

A greater surface area of leaf litter and cotton substrate in contact with sediment microorganisms when buried, and generally oxic hyporheic water in our gravel-dominated study streams, may have also contributed to greater C processing in the hyporheic zone compared to surface patches. Although dissolved oxygen is often a limiting resource for hyporheic macroinvertebrate and aerobic microbial colonisation and activity^19,23,25^, coarse-gravel material combined with short hydrological residence times has been associated with oxygen-rich hyporheic water^26,27^, and may have sustained the high rates of microbial activity in our study. It is possible that in sand or silt-dominated intermittent stream channels, lower hydraulic conductivity may lead to anoxic sediments and thus lower rates of particulate C processing in the hyporheic zone than we observed in this study. Nonetheless, the ubiquitous patterns in hyporheic C processing that we observed indicate that dissolved oxygen, although important, is likely a secondary driver compared to the role of substrate saturation in gravel-dominated intermittent streams.

The rates of surface and hyporheic leaf processing recorded in our study were greater than comparable studies elsewhere, even when more palatable leaf species were used (Table 1). This finding was unexpected given that eucalypt leaves are generally consumed more slowly than many other leaf types due to their toughness, high concentrations of secondary metabolites, tannins and phenolic molecules as well as low nitrogen concentrations^28,29^. However, the relatively high rates of eucalypt leaf processing that we recorded corroborates findings from a gravel and cobble dominated stream in south-eastern Australia^20^, and may suggest that other environmental factors (i.e. temperature, oxygen concentration, presence of bacteria and fungi) may negate issues related to reduced substrate palatability of eucalypt leaves. Multiple interacting stressors can increase leaf breakdown rates^30^. Indeed, surface and hyporheic water temperature and hyporheic oxygen concentrations recorded in our study were generally greater than in comparable studies (see Table 1), and these conditions possibly contributed to the relatively high rates of leaf processing we observed. Moreover, the fact that we recorded similar rates of microbial respiration on eucalypt leaves and cotton strips (except for surface patches of gravel bars), which are two substrates with different C molecular structure and thus perceived biotic palatability (cotton is predominately cellulose with minimal organic acids^31^), indicate that microbial activity on eucalypt leaves was not negatively impacted by its perceived lower palatability.

Surface organic C processing was primarily controlled by water availability, with leaf litter and cotton processing strongly related to benthic substrate saturation. In particular, cotton processing was more than 7.5 times greater in saturated than in dry patches, irrespective of their location. Furthermore, both leaf litter and cotton processing were greatest in perennial compared to intermittent active-channel patches (i.e. pools and riffles). The degree of immersion has previously been documented to be a major factor controlling detrital processes in intermittent^32–35^ and perennial^21,36^ streams. For example, a decrease in flow permanence from 100 to 85% led to a four-fold decrease in leaf litter breakdown in a temporary stream in south-west France^8^. Additionally, leaf (*Populus* sp.) breakdown rates decreased with increasing cumulative emersed duration (i.e., total number of days of emersion during the experiment) in a New Zealand temporary river^37^. Our findings, and the previous research, indicate that small reductions in flow permanence may have large impacts on organic C processes in wetted stream environments.

Our research highlights the critically important role of the hyporheic zone of intermittent streams for organic C processes, even when surface flows cease. This adds to a small but growing body of literature demonstrating the ecological and biogeochemical importance of dry stream channels^6,7,38,39^. Given that the hyporheic zone often remains saturated during dry periods^12^, and that intermittent streams constitute 69% of first order streams below 60 °N^4^, conditions that maintain high rates of hyporheic organic C processing are likely prevalent in many catchments. Research is thus required to better understand how sub-surface flow-paths and substrate saturation varies across intermittent stream networks and over time and what consequences this has for landscape C processes. Finally, sediment saturation was the key factor underpinning the sustained and high rates of organic C processing. Consequently, human-induced changes that reduce baseflow in intermittent streams and rivers, such as groundwater extraction and reduced regional rainfall due to climate change, may lead to large reductions in the rates of organic C processing in intermittent stream channels.

## Methods

### Study area and design

Eight streams were selected from two regions located in humid subtropical^40^ eastern Australia (Supplementary Fig. S4). Two regions were chosen to broaden the potential transferability of the findings. During the study period, precipitation displayed no strong seasonal patterns but air temperature peaked during summer (Supplementary Fig. S5). Five streams were situated in south-eastern Queensland (QLD) and three streams in northern New South Wales (NSW). Gravel, cobble, and rock substrate dominates surface and hyporheic patches in the streams, particularly in south-eastern QLD (Supplementary Information Table S1). A perennial study stream was included in the NSW region (Horsearm Creek), with all other study streams in both regions experiencing intermittent surface flow. Hyporheic exchange was spatially and temporally variable within each study stream, but could broadly be categorised as downwelling, neutral, or upwelling (Table 2; see Supplementary Methods for hyporheic exchange methodology).

Particulate organic C processing experiments were undertaken within surface and hyporheic patches of the permanently connected channel (pools and riffles) and the parafluvial zone (gravel bar) at multiple times, spanning temporal changes in surface water extent, substrate saturation, and temperature. We randomly chose (using a random number table based on stream dimensions) five replicate patches of each surface channel unit type for the experimental assessment of organic C processing experiments and measurement of substrate moisture (see below). We hammered a steel bar into the substrate to attach all experimental substrates with cable ties during incubations. Hyporheic deployments occurred approximately 1 m downstream of the paired surface patches at a depth of 30 ± 5 cm below the surface. We buried experimental organic C substrates (leaf litter bags and cotton strip assays – see below) following the procedure of ref.^41^ by excavating sediment within a contained temporary cylinder to divert the water current and minimise disturbance. HOBO pendant temperature loggers (Onset, Bourne, U.S.A) were placed alongside a subset of leaf litter bags at each site in order to assess the influence of water temperature on rates of C processing. Temperature was recorded every 30 minutes.

### Organic C processing experiments

We quantified rates of particulate organic C processing using replicate fine- and coarse-mesh leaf litter bags and cotton strip assays. These techniques quantify leaf processing (% leaf mass loss and decay rate) and loss of cotton tensile strength, respectively, and are widely used for assessing organic C processing^42–44^. In the QLD region, leaf litter bags were deployed twice (September to November 2015 and February to April 2016) and cotton strip assays four times (November 2015, January/February 2016, February/March 2016, and April/May 2016). In the NSW region, both leaf litter bags (September to November 2015 and March to May 2016) and cotton strip assays (September to October 2015 and March to April 2016) were deployed twice.

We collected freshly-senesced eucalypt leaves with minimal evidence of disease or blemishes from the dominant riparian tree species within each region (*Eucalyptus tereticornis* in QLD and *E. camaldulensis* in NSW) for use in leaf litter bags. Approximately 3 g ( ± 0.05 g) of oven-dried (60 °C for 48 hours) leaves with stalks removed were placed into fine-mesh (0.5mm mesh-size, width = 15 cm, length = 15 cm) and coarse-mesh (5mm mesh-size, width = 12 cm, length = 17 cm) bags. Variation in leaf litter processing between fine- and coarse-mesh bags is commonly used to differentiate microbial (bacteria and fungi) from macroinvertebrate leaf consumption because fine-mesh bags exclude macroinvertebrates^43^. We deployed one fine-mesh and one coarse-mesh leaf litter bag in each paired surface and excavated hyporheic patch (see ‘Study area and design’ for a more thorough description of the experimental design), leading to five replicates for each depth in each channel unit type (pools, riffles, gravel bars) in each stream. Leaf bags were collected after a minimum of 60 days. Upon collection, samples were placed into labelled sealed bags and frozen within 24 hours. In the laboratory, samples were defrosted, rinsed, and dried at 60 °C for 48 hours. The dry weight was recorded and samples were then combusted at 550 °C for 40 minutes to obtain ash weight. The remaining litter ash-free dry mass (AFDM) was then calculated as the difference between dry and ashed weight remaining. Similarly, control leaves (to calculate the initial litter AFDM) and leaves leached for 24 hours (to account for mass loss due to leaching) were processed as above.

The percent leaf mass loss was calculated using the following equation:

$$ \% \,Mas{s}_{loss}\,=\,AFD{M}_{loss}/AFD{M}_{initial}\,\times \,100$$

As the duration of leaf litter deployments differed among sites and regions (between 60 and 70 days), values of leaf litter % mass loss were scaled to an incubation period of 60 days prior to data analyses by dividing each leaf litter % mass loss value by the number of incubation days and multiplying by 60. Scaling to degree days was not possible for leaf litter processing (and cotton strip decay) as cycles and wetting and drying in intermittent streams prevent the continuous recording of water temperature. Leaf litter decay rates (*k* coefficients) were calculated, to directly compare rates of leaf litter processing with studies in other regions, using a negative exponential decay model:

$$k={ln}(\frac{{M}_{t}}{{M}_{o}})\div t$$

where *M*_*t*_ is the AFDM at time *t* (i.e. AFDM remaining) and *M*_*o*_ is the initial AFDM. This equation assumes that the mass loss rates follow an exponential pattern.

Cotton strip assays provide a standardised measure of particulate organic C processing because cotton is 95% cellulose and breakdown rates are thus not confounded by variation in chemical composition, as can be the case when assessing decomposition with leaves^42,44^. Unbleached and unprimed cotton fabric was used for assays in all streams. Two replicate cotton strips (35mm by 60mm), affixed to a plastic ruler with rubber bands and cable ties, were placed cotton-side-up on the bottom of each surface or excavated hyporheic patch at the beginning of each incubation period. After approximately 28 days, the rulers and strips were collected and rinsed, if necessary, to remove deposited sediment. Within 5 hours of collection, the cotton strips were removed from the rulers and placed on paper to air-dry before being dried at 40 °C for 24 hours in the laboratory. Once strips were dry, the tensile strength of each was measured with a Digital Force Gauges Series 4 tensiometer (Mark-10, New York, U.S.A.) at the Water Research Laboratory, UNSW Sydney. Cotton tensile strength (kilogram-force or kgf) was recorded as the initial breaking point of each strip. Ten randomly selected cotton strips were used as procedural controls to determine the mean and standard deviation of pre-incubation cotton tensile strength (50.03 ± 2.8 kgf). The data was represented as the cotton tensile strength loss (CTSL) by subtracting the cotton tensile strength of procedural controls with that of incubated strips. Greater CTSL values represent a higher particulate organic C processing than values closer to zero. As the incubation periods differed slightly among sites and regions (27 to 44 days), and cotton breakdown is linear^42^, CTSL values were scaled to an incubation period of 28 days prior to data analysis by dividing each CTSL value by the number of incubation days and multiplying by 28. Replicate cotton strips in each surface or excavated hyporheic patch were averaged prior to statistical analyses, leading to five replicate values per patch in each stream.

### Microbial respiration experiments

We also investigated patterns in the rates of microbial respiration (MR) of heterotrophic biofilms that colonised leaf (*E. tereticornis*) and artificial cellulose substrates in the QLD study sites. Leaves and 9 cm^2^ artificial cellulose substrates, made from Vileda sponge cloth (Freudenberg Household Products, Weinheim, Germany), were placed in fine-mesh bags alongside the leaf litter and cotton strip experiments in surface and hyporheic patches of gravel bars, riffles, and pools (i.e. five replicate sponge and leaf substrates for each patch per stream). Substrates were collected after 28 days and respiration was measured *in-situ* (within 1 hour) using the modified dark-chamber method^45^ following the procedure in ref.^46^. A detailed description of the MR experimental procedure is available in the Supplementary Methods. MR was calculated as the differences in O_2_ between start and finish of a three hour incubation, correcting for background O_2_ consumed from bacterioplankton. MR was represented per gram of incubated substrate AFDM per hour (as mg O_2_ consumed g^−1^ AFDM h^−1^).

### Environmental variables

We characterised substrate moisture using an ordinal measure derived from the substrate moisture status (dry, moist, or saturated) at the start and end of the deployment period: Dry-Dry = 1, Dry-Moist = 2, Moist-Dry = 2, Dry-Saturated = 3, Saturated-Dry = 3, Moist-Moist = 4, Moist-Saturated = 5, Saturated-Moist = 5, and Saturated-Saturated = 6. Patches with higher substrate moisture values were assumed to be saturated for a longer period than those with lower values. It was impossible to assess changes in patch-scale flow or moisture conditions using traditional methods, such as using v-notch weirs and a flow hydrograph, due to the braided channel morphology, intermittent flow, and dynamic patterns in surface-hyporheic connectivity that led to patchy surface water persistence and flow paths within a stream reach. Dissolved oxygen and pH were measured in representative surface (submerged patches only) and hyporheic (only DO) patches using a HQ40d portable meter (HACH, Loveland, U.S.A.) when water was present. We collected water samples from riffles, or pools when riffles were absent, to measure ambient concentrations of dissolved organic carbon (DOC), nitrate (NO_3_^−^), ammonium (NH_4_^+^), and soluble reactive phosphorus (SRP). Water samples were filtered in the field (0.45 µm nylon membrane filters; Sarstedt) and transferred to a freezer within 6 hours. These water samples were not collected on all sampling occasions. DOC was analysed with the combustion catalytic oxidation method (method APHA 5310D). NO_3_^−^, NH_4_^+^, and SRP were analysed with a SEAL Analytical AutoAnalyzer 3 (Porvair Sciences, Wrexham, UK). Mean riffle and pool depth was calculated from several measurements made at random locations at times of surface flow. Interactions between groundwater and surface water were examined using ^222^Rn activity measurements in surface water. ^222^Rn is an excellent tracer to identify areas of significant groundwater influence because groundwater is very enriched in ^222^Rn compared to surface waters (typically 1000-fold or greater), it is chemically unreactive, volatile and has a short half-life (t_1/2_ = 3.83 d)^47^. A detailed description of the collection and analysis of samples for ^222^Rn is available in the Supplementary Methods.

### Statistical analyses

We used a linear mixed-effects model (LMM) to assess differences in the % leaf mass loss among fine- and coarse-mesh bags for both surface and hyporheic environments, with mesh size, and channel unit as crossed fixed factors, and study site was a random variable. If no significant differences were evident among mesh sizes they were combined for subsequent analyses. LMMs were also used to assess differences in the % leaf mass loss and CTSL among depths and channel units. We performed separate LMMs for each study region with depth and channel unit as fixed factors and experiment patch (nested within incubation period), incubation period and study site as random factors. We performed separate models for each region due to a) the different leaf species used, b) differences in the number of cotton strip assays in each region, and c) to broaden the transferability of the findings. All models including channel unit as a factor distinguished riffle and pool channel units in intermittent streams from those in the perennial study site. We combined % leaf mass loss and CTSL data in gravel bars for intermittent and perennial patches because prior data exploration revealed they were similar. We used separate LMMs to assess differences in leaf and cellulose biofilm MR among depths and channel units (fixed factors), with site and incubation period (for cellulose substrates) as random variables. We assessed difference in biofilm MR among leaf and cellulose substrates in each channel unit (fixed factors) within the first incubation period (common to both substrates) using separate LMMs for each depth (surface and hyporheic), with replicate patch nested within site as the random factor. The relationship between surface and hyporheic values for % leaf-mass loss, CTSL and MR on leaf and cellulose substrate with substrate moisture status (ordinal numerical factor) was investigated using LMMs, with site and/or incubation period nested within region as random factors. Linear regression was used to assess the association of water temperature with % leaf-mass loss and leaf decay rate (*k*) using only those leaf packs which remained saturated for the entire incubation period (i.e. substrate moisture status = 6) and which had a corresponding temperature logger. We assessed variability in leaf decay rates among surface and hyporheic environments of intermittent and the perennial stream by calculating the coefficient of variation. We assessed differences in substrate moisture among depths (fixed factor) using a LMM, with depth nested within site (random factor). Restricted maximum likelihood was used to estimate the variance components, and *P* values and degrees of freedom were estimated with log likelihood ratio tests for LMMs^48^. We used Tukey’s honest significance difference (HSD) test for pairwise comparisons among means. We considered results significant if *P* ≤ 0.05. LMMs were performed with the “lme4” R package and were, along with all other analyses, conducted in R^49^.

### Data availability

The datasets generated during and/or analysed during the current study are available from the corresponding author on reasonable request.

## Electronic supplementary material

Supplementary Information
