# Supplementary material for: High rates of organic carbon processing in the hyporheic zone of intermittent streams
Source: Sci Rep. 2017 Oct 16;7:13198. doi: 10.1038/s41598-017-12957-5 (PMC5643499; doi:10.1038/s41598-017-12957-5)
Supplement: Supplementary file 1 — Supplementary Information [file 41598_2017_12957_MOESM1_ESM.pdf]

Supplementary Information

Title: High rates of organic carbon processing in the hyporheic zone of intermittent streams

Authors: *Ryan M. Burrows[1], Helen Rutlidge[2,3], Nick Bond[1,4], Stefan Eberhard[2,5], Alexandra Auhl[2,6], Martin S. Andersen[2,3], Dominic Valdez[1], Mark J. Kennard[1].

[1]Australian Rivers Institute, Griffith University, Nathan, Queensland, Australia.

[2]Connected Waters Initiative Research Centre, UNSW Sydney, Australia.

[3]School of Civil and Environmental Engineering, UNSW Sydney, Australia.

[4]Murray-Darling Freshwater Research Centre, La Trobe University, Wodonga, Australia.

[5]Subterranean Ecology Pty Ltd, 227 Coningham Road, Coningham, Tasmania, Australia.

[6]School of Biological, Earth and Environmental Sciences, UNSW Sydney, Australia.

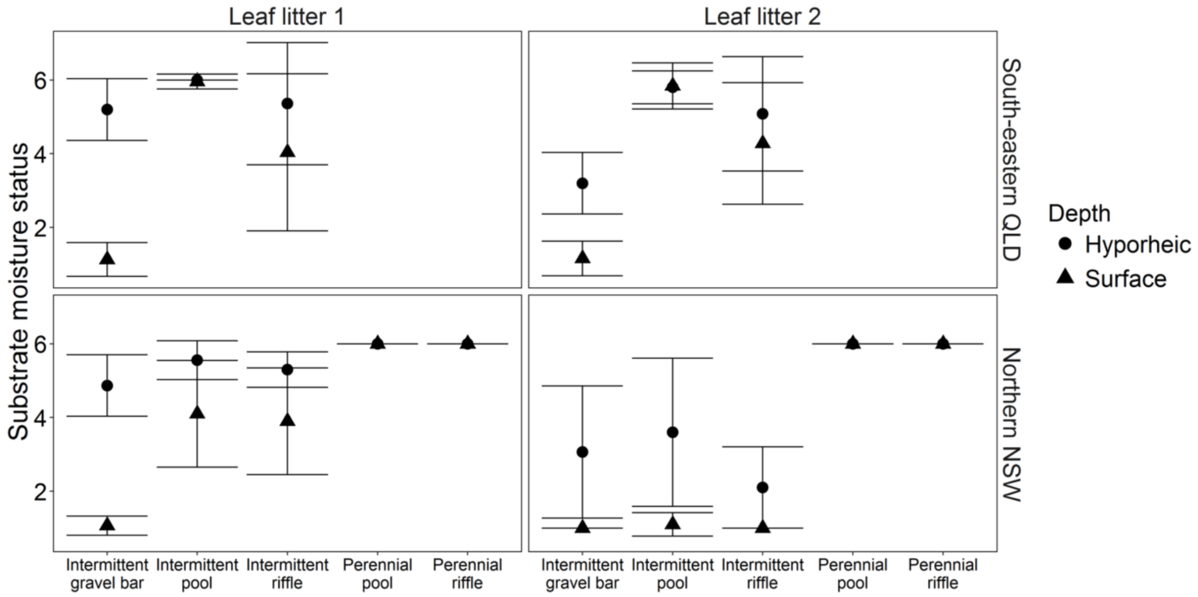

Supplementary Fig. S1. The mean and standard deviation of substrate moisture status in the surface and hyporheic zone of each channel unit and each leaf litter incubation period (Leaf 1 and 2).

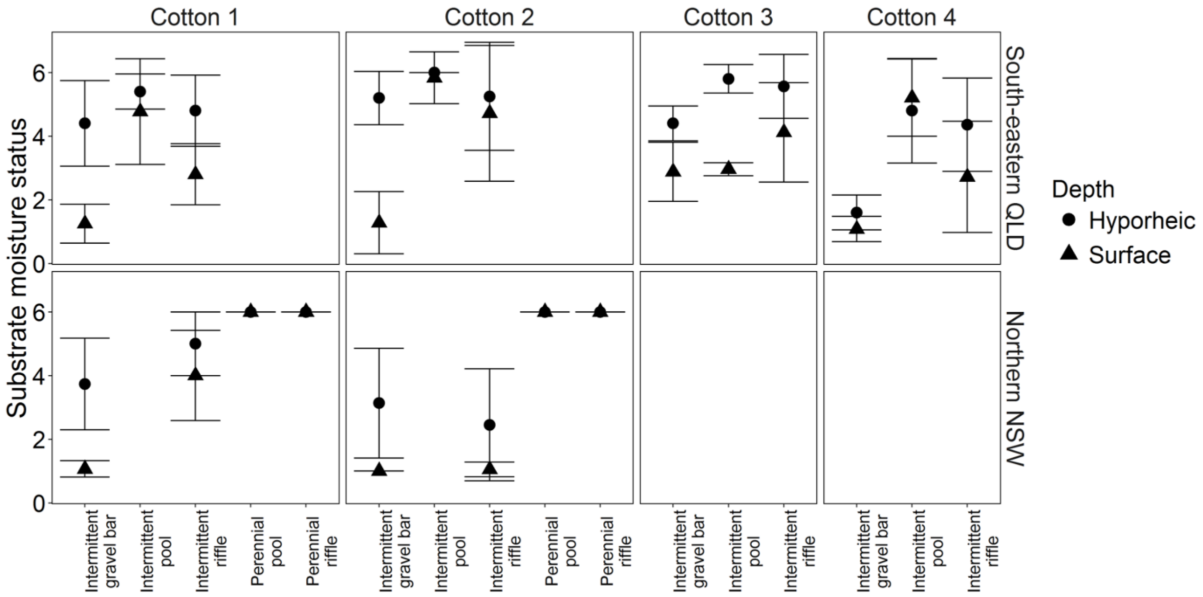

Supplementary Fig. S2. The mean and standard deviation of substrate moisture status in the surface and hyporheic zone of each channel unit and each cotton strip incubation period (Cotton 1 – Cotton 4).

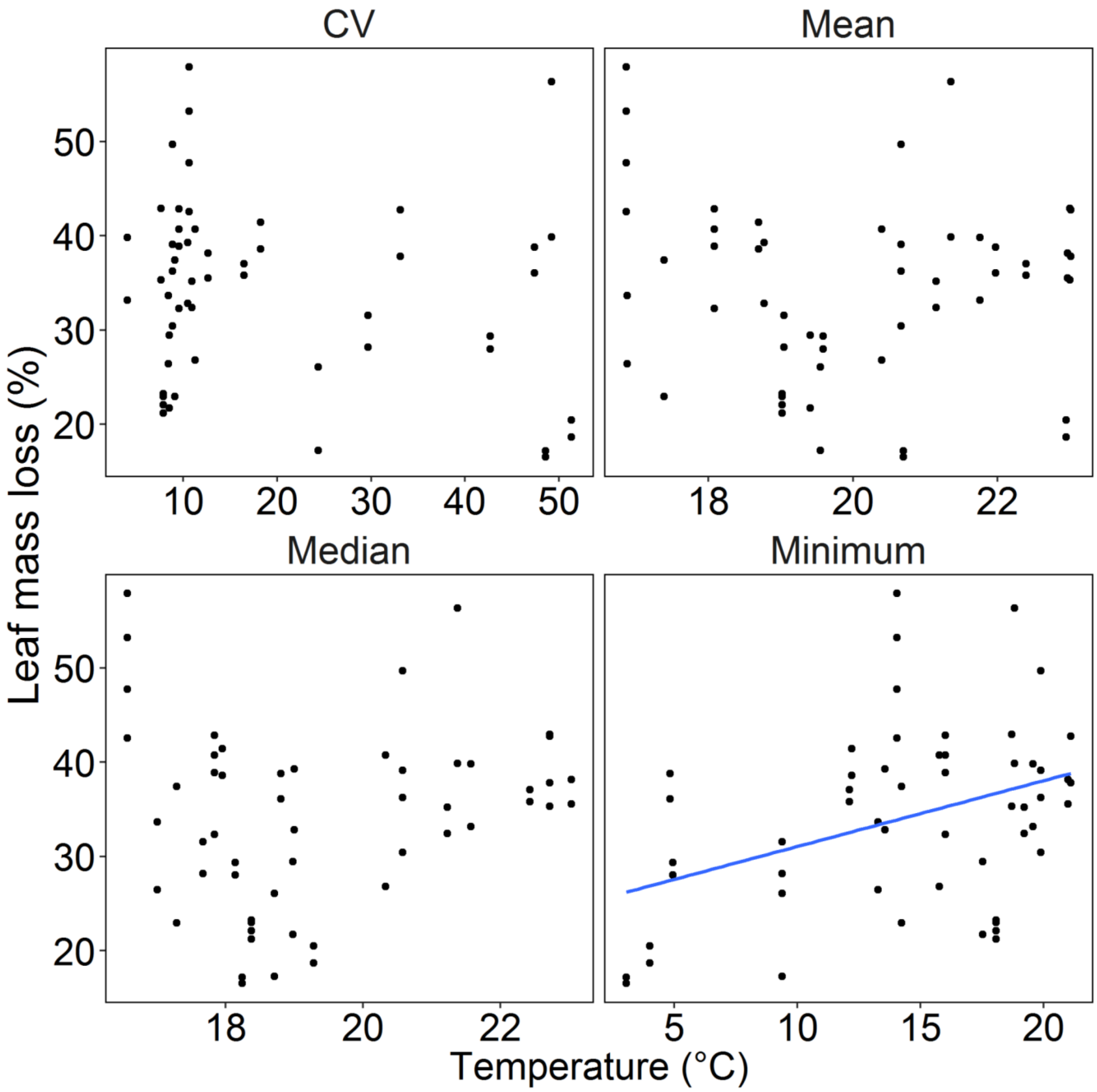

Supplementary Fig. S3. The relationship between mean, median, minimum, and coefficient of variation (CV) of water temperature (°C) with leaf mass loss (%). A line of best fit is displayed for the significant linear association between minimum water temperature and leaf mass loss (R^2=0.15, P=0.004, n=52).

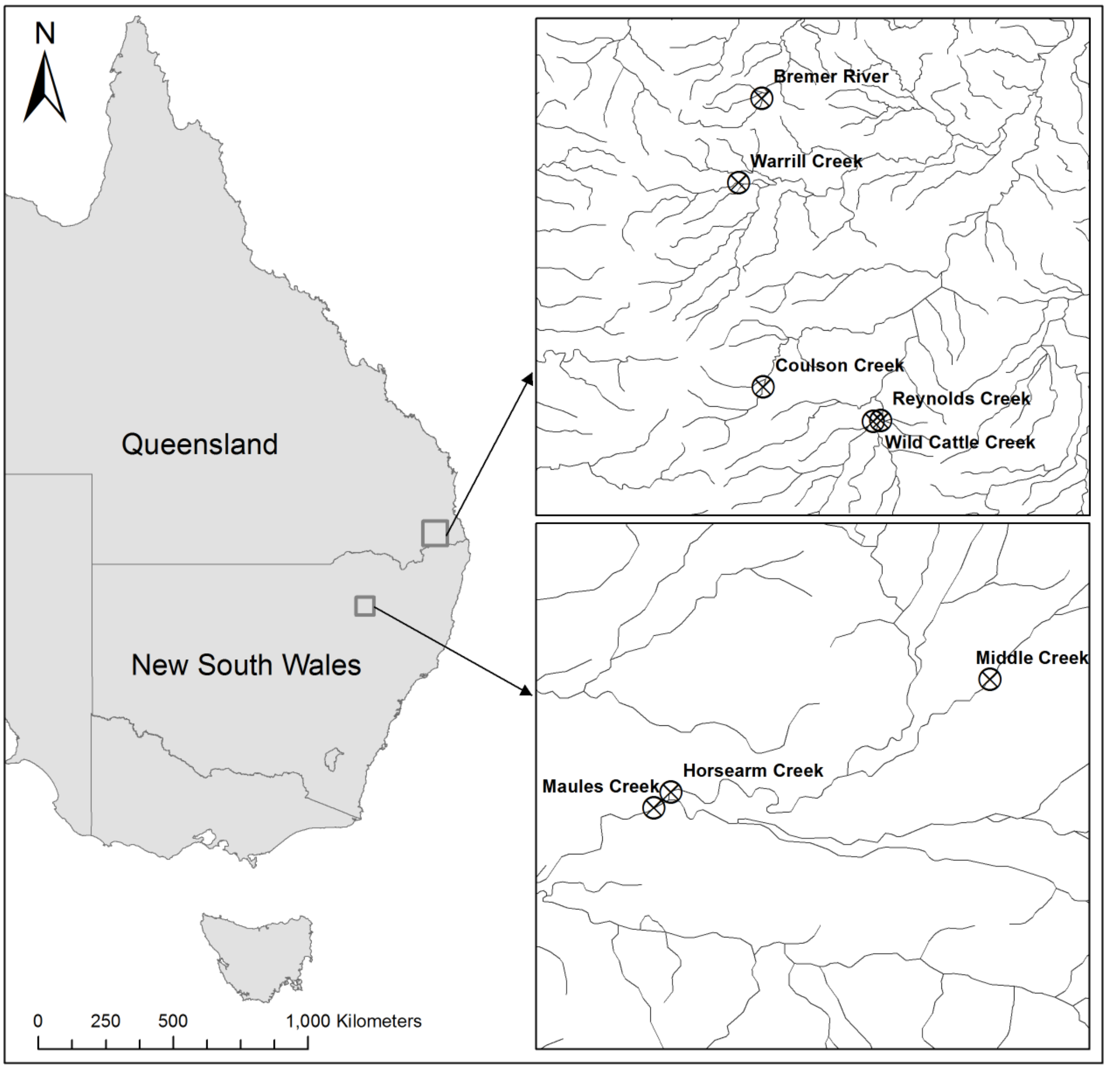

Supplementary Fig. S4. Location of the eight study streams in eastern Australia. This map was produced using ArcMap (version 10.3 ; http://www.desktop.arcgis.com) with the stream channel network sourced from the Australian Hydrological Geospatial Fabric (Geofabric; http://www.bom.gov.au/water/geofabric/).

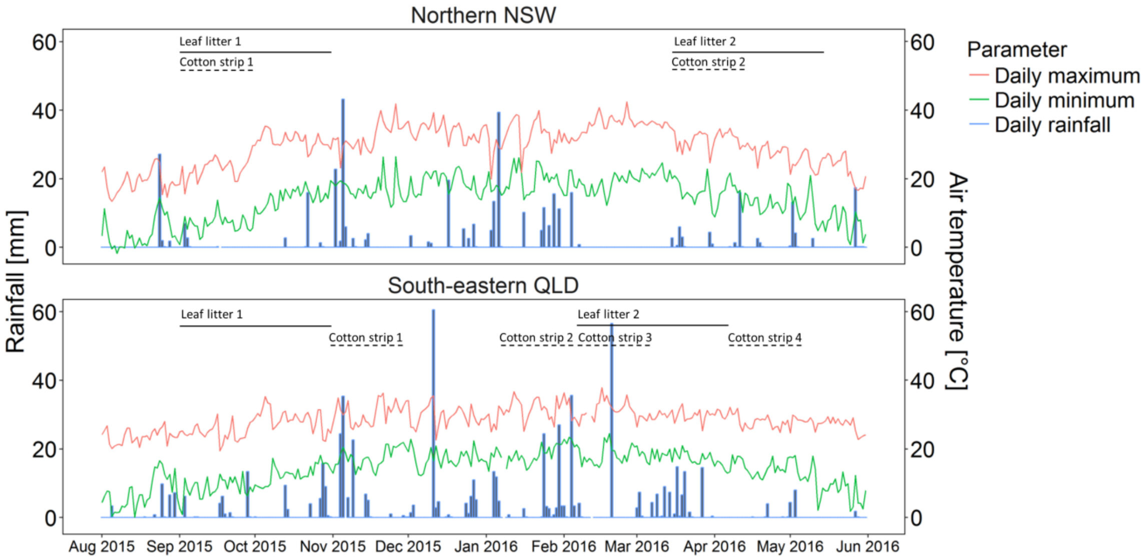

Supplementary Fig. S5. The daily rainfall (mm) and daily minimum and maximum air temperature (°C) experienced in each study region. Rainfall and air temperature data are from the Australian Bureau of Meteorology [accessible at: http://www.bom.gov.au/?ref=logo] from weather stations at Narrabri Airport (054038) for northern New South Wales (NSW) and Beaudesert (040983) for south-eastern Queensland (QLD). The periods for leaf litter and cotton strip incubations are overlaid.

Supplementary Table S1. The mean (± standard deviation) proportion of each substrate type contained within surface and hyporheic patches in each study stream. There are five replicate patches within each channel unit (gravel bar, riffle, and pool). Surveys were completed in the south-eastern Queensland streams (Bremer River, Coulson Creek, Reynolds Creek, Warrill Creek, and Wild Cattle Creek) between 1st and 4th September 2015. Surveys were completed in the northern New South Wales streams (Horsearm Creek, Maules Creek, and Middle Creek) between 1st and 3rd September 2015 (hyporheic only). Substrate composition was visually estimated (% of patch surface area, approximately 30cm diameter circle) using a modified Wentworth scheme (Wentworth 1922). Mud/clay<0.06 mm; sand=0.06-2.0 mm; fine gravel=2.1-16.0 mm; coarse gravel=16.1-64.0 mm; cobble=64.1-128.0 mm; rock=128.1-512 mm; bedrock>512.1 mm.

*Wentworth, C. K. 1922. A Scale of Grade and Class Terms for Clastic Sediments. The Journal of Geology 30: 377-392.

Site	Depth	Mud/Clay	Sand	Fine gravel	Coarse gravel	Cobble	Rock	Bedrock	CPOM
Bremer River	Hyporheic	2.80 (2.17)	2.00 (2.74)	5.60 (3.21)	4.20 (4.92)	31.00 (18.17)	30.40 (17.20)	0.00	24.00 (8.94)
Bremer River	Surface	0.13 (0.52)	1.33(3.99)	4.13 (4.37)	14.93 (12.31)	31.47 (25.91)	15.87 (16.56)	0.00	32.13 (19.50)
Coulson Creek	Hyporheic	0.00	0.00	2.20 (2.28)	14.20 (12.34)	35.00 (7.91)	17.60 (11.89)	0.00	31.00 (10.65)
Coulson Creek	Surface	1.13 (2.47)	2.20 (4.52)	7.67 (10.88)	12.47 (10.51)	26.00 (18.34)	14.80 (12.58)	10.73 (25.15)	25.00 (16.63)
Reynolds Creek	Hyporheic	1.20 (1.79)	0.00	5.20 (2.17)	13.2 (3.70)	63.80 (7.46)	9.00 (8.94)	0.00	7.60 (3.65)
Reynolds Creek	Surface	1.67 (4.50)	0.47 (1.25)	5.07 (4.18)	14.6 (9.74)	50.13 (18.01)	12.87 (10.37)	0.00	15.20 (21.63)
Warrill Creek	Hyporheic	0.00	0.53 (2.07)	29.70 (39.15)	22.83 (29.98)	30.53 (35.40)	9.67 (18.75)	0.00	6.73 (9.57)
Warrill Creek	Surface	0.00	1.33 (3.99)	28.10 (36.94)	22.27 (27.48)	31.27 (37.65)	7.67 (12.66)	0.00	9.37 (11.98)
Wild Cattle Creek	Hyporheic	0.00	0.00	5.40 (3.85)	16.20 (4.44)	52.60 (27.43)	18.00 (24.90)	0.00	7.80 (1.92)
Wild Cattle Creek	Surface	3.67(11.72)	0.00	11.40 (12.37)	19.47 (15.92)	44.93 (23.09)	12.13 (18.47)	0.00	8.40 (11.11)
Horsearm Creek	Hyporheic	6.33 (22.18)	20.53 (18.63)	21.53 (19.33)	20.80 (19.91)	7.00 (12.62)	0.67 (2.58)	0.00	23.13 (22.44)
Horsearm Creek	Surface	NA	NA	NA	NA	NA	NA	NA	NA
Maules Creek	Hyporheic	0.33 (1.29)	15.00 (15.35)	28.33 (10.29)	37.00 (16.99)	13.33 (12.77)	1.00 (2.80)	0.00	5.00 (5.00)
Maules Creek	Surface	NA	NA	NA	NA	NA	NA	NA	NA
Middle Creek	Hyporheic	0.00	22.60 (28.73)	18.13 (12.08)	26.67 (18.58)	16.60 (19.23)	13.00 (18.88)	0.00	3.00 (3.34)
Middle Creek	Surface	NA	NA	NA	NA	NA	NA	NA	NA

Supplementary Methods

Hyporheic exchange methodology

At each sampling location, vertical hydraulic head difference (VHH) measurements were taken to allow each location to be classified as upwelling, neutral, down-welling, or dry using temporary installed piezometers. Between three and six temporary piezometers were installed at random intervals along the length of each stream reach. VHH measurements were made twice, corresponding with wetter and drier flow conditions in each region. In south-eastern QLD, dry-season sampling was conducted between 3-7th September 2015 and wet-season sampling between 7-10th March 2016. In northern NSW, wet-season sampling was conducted between 21st-23rd July 2015 and dry-season sampling between 5-6th April 2016. At some locations the VHH results were ambiguous, potentially due to the temporary piezometers not having enough time to recover especially in low-permeability sediments. Therefore, Partial Least Squares (PLS) regression was applied to a subset of the dataset with reliable VHH measurements to determine if there was a physico-chemical signature related to upwelling or down-welling which could be applied to the remaining dataset. The measured VHH, along with the results from PLS and hydrological conditions, were then used to classify each sample as either hyporheic upwelling, neutral, downwelling, or dry.

Method for measuring microbial respiration using a dark-chamber method

Microbial respiration of incubated leaf and artificial cellulose substrates was assessed using a modified dark-chamber method. At each surface and hyporheic patch, fine-mesh bags containing leaf and artificial cellulose substrates were carefully removed. Substrates were removed from each the fine-mesh bag and placed in 50mL Falcon centrifuge tubes (Corning Incorporated, Corning, NY, U.S.A) filled with unfiltered stream water. After all substrates were transferred to centrifuge tubes, stream water in each centrifuge tube was discarded and

replaced with freshly collected unfiltered stream water of known dissolved oxygen concentration measured using a handheld O_2 meter (YSI, Yellow Springs, OH, U.S.A). Each centrifuge tube was capped underwater, ensuring that air bubbles were not present, and then incubated in stream water at each site in darkness (covered by a black plastic container) for 3 hours. Along with the centrifuge tubes containing leaf and artificial cellulose substrates, three centrifuge tubes during each incubation were filled with only unfiltered water from each site to correct for background changes in O_2 due to bacterioplankton activity throughout the incubation. At the end of the 3 hour incubation period, centrifuge tub caps were removed and a final O_2 measurement was taken. Microbial respiration was calculated as the differences in O_2 between start and finish of a 3-hour incubation, correcting for background O_2 consumed from bacterioplankton. Microbial respiration was represented per gram of incubated substrate AFDM per hour (as mg O_2 consumed g^{-1} AFDM h^{-1}). Leaves were deployed once (January/February 2016) and artificial cellulose substrates three times (January/February 2016, February/March 2016, and April/May 2016).

Collection and analysis of samples for ^{222}Rn

Three replicate two liter surface water samples, with zero headspace, were collected from each stream during a period of surface-water flow. Within 24 hours, ^{222}Rn (becquerels per cubic meter; Bq m^{-3}) within these water samples were measured using a RAD-7 radon detector (DURRIDGE Company Inc., Billerica, U.S.A.) following the methods in Burnett & Dulaiova (2003). Final ^{222}Rn values were back-calculated to account for radioactive decay from time of sampling.

Burnett, W. C. & Dulaiova, H. Estimating the dynamics of groundwater input into the coastal zone via continuous radon-^{222} measurements. *Journal of Environmental Radioactivity*. **69**: 21-35 (2003).
